# Multi-omics approaches for biomarker discovery and precision diagnosis of prediabetes

**DOI:** 10.3389/fendo.2025.1520436

**Published:** 2025-03-14

**Authors:** Jielin Song, Chuanfu Wang, Tong Zhao, Yu Zhang, Jixiang Xing, Xuelian Zhao, Yunsha Zhang, Zhaohui Zhang

**Affiliations:** ^1^ Graduate School, Tianjin University of Traditional Chinese Medicine, Tianjin, China; ^2^ TCM Institute of Sore and Ulcer, Tianjin University of Traditional Chinese Medicine, Tianjin, China; ^3^ Tianjin Institute of Traditional Chinese Medicine Surgery, Tianjin, China; ^4^ Department of Encephalopathy, Liangping District Hospital of Traditional Chinese Medicine, Chongqing, China; ^5^ School of Integrative Medicine, Tianjin University of Traditional Chinese Medicine, Tianjin, China; ^6^ Department of Traditional Chinese Medicine Surgery, The Second Affiliated Hospital of Tianjin University of Traditional Chinese Medicine, Tianjin, China

**Keywords:** prediabetes, multi-omics, biomarkers, diagnosis, management, data integration

## Abstract

Recent advancements in multi-omics technologies have provided unprecedented opportunities to identify biomarkers associated with prediabetes, offering novel insights into its diagnosis and management. This review synthesizes the latest findings on prediabetes from multiple omics domains, including genomics, epigenomics, transcriptomics, proteomics, metabolomics, microbiomics, and radiomics. We explore how these technologies elucidate the molecular and cellular mechanisms underlying prediabetes and analyze potential biomarkers with predictive value in disease progression. Integrating multi-omics data helps address the limitations of traditional diagnostic methods, enabling early detection, personalized interventions, and improved patient outcomes. However, challenges such as data integration, standardization, and clinical validation and translation remain to be resolved. Future research leveraging artificial intelligence and machine learning is expected to further enhance the predictive power of multi-omics technologies, contributing to the precision diagnosis and tailored management of prediabetes.

## 1 Introduction

Diabetes has emerged as a critical global public health issue, with its prevalence nearly doubling over the past three decades and continuing to rise unabated (1, 2). The International Diabetes Federation reported that approximately 537 million adults (aged 20–79) were living with diabetes in 2021, with this number expected to reach 643 million by 2030 and 783 million by 2045 (3). Diabetes is characterized by numerous complications, substantial healthcare expenditures, and high mortality, ranking it among the top ten global causes of death. It not only reduces the quality of life for patients but also imposes significant economic and societal burdens (4–6). Age and disease duration are key predictors of diabetes management outcomes, underscoring the importance of early diagnosis and timely intervention in prediabetes (7).

Prediabetes is defined as an intermediate metabolic state where blood glucose levels are elevated but not yet meet the diagnostic thresholds for diabetes. It encompasses impaired fasting glucose (IFG), impaired glucose tolerance (IGT), or both. The global prevalence of prediabetes is also on the rise, with approximately 373.9 million individuals (7.5%) affected in 2019. This figure is projected to reach 453.8 million (8.0%) by 2030 and 548.4 million (8.6%) by 2045 (8). Prediabetes is often asymptomatic, and studies suggest that approximately 50% of individuals with diabetes are unaware of their condition (8). Prediabetes constitutes a high-risk state, with approximately 70% of individuals progressing to diabetes over time (9, 10). Many individuals may already have macrovascular complications at the time of diabetes diagnosis. Moreover, prediabetes substantially increases the risk of cardiovascular disease and stroke, challenging its classification as a benign condition (11, 12). Lifestyle interventions in individuals with prediabetes have been shown to reduce the risk of diabetes by 40% to 70%. Early detection of prediabetes is critical to extending the intervention window and improving disease management, ultimately reducing morbidity, complications, and premature mortality.

## 2 Pathophysiological mechanisms underlying prediabetes

Compared to type 2 diabetes (T2D), blood glucose levels in type 1 diabetes mellitus rise more rapidly, presenting as a more acute and severe condition. Consequently, clinicians have more time to detect glucose abnormalities associated with prediabetes in T2D (13). The pathophysiology of prediabetes shares substantial overlap with T2D, characterized by insulin resistance (IR) and β-cell dysfunction. IR leads to increased insulin demand, and when β-cells fail to compensate, glucose levels begin to rise, marking the transition from normoglycemia to prediabetes and eventually T2D (14–16).

β-cell dysfunction is a critical feature in the progression of T2D, often manifesting years before the clinical diagnosis. In individuals with severe IR, plasma glucose levels may remain within the normal range as long as β-cell compensation is adequate. Conversely, prediabetes emerges when β-cells fail to secrete sufficient insulin to maintain normal glucose levels. Timely interventions targeting β-cell function can reverse or delay the progression toward T2D in individuals at risk (17).

Weir et al. proposed a three-stage model of diabetes development. In the first stage, IR arises, accompanied by increased insulin secretion and β-cell mass. As long as insulin secretion compensates for IR, glucose levels remain within the normal range. In the second stage, β-cell adaptation becomes insufficient to fully compensate for the growing IR, resulting in mild hyperglycemia, reflected in elevated fasting or post-load glucose levels, which are hallmarks of prediabetes. The third stage marks early decompensation, where β-cells can no longer meet the body’s insulin demands, causing glucose levels to rise rapidly and leading to overt diabetes (16, 18).

Although individuals with IFG and IGT both exhibit IR, the distribution of IR differs. Hepatic IR is predominant in individuals with IFG, while skeletal muscle IR is relatively uncommon (18–20). In contrast, IGT is primarily associated with skeletal muscle IR, with minimal changes in hepatic insulin sensitivity (20, 21). The decline in insulin sensitivity is progressive, transitioning from normal glucose tolerance (NGT) to IFG, IGT, and eventually T2D. Both IFG and IGT are accompanied by β-cell dysfunction, with early-stage insulin secretion deficits observed in both conditions. Notably, individuals with IGT also exhibit impaired insulin secretion during the later stages of glucose intolerance (21).

Emerging evidence suggests that chronic low-grade inflammation and adipose tissue dysfunction also play pivotal roles in the development of prediabetes and T2D (22, 23). Dysregulated adipokine secretion and ectopic fat deposition, such as hepatic steatosis, further exacerbate IR and β-cell dysfunction (22, 24). These additional factors highlight the complexity of the pathophysiological processes underlying prediabetes, underscoring the need for multifaceted therapeutic approaches targeting both metabolic and inflammatory pathways.

## 3 The current status of prediabetes diagnosis

The diagnostic criteria for prediabetes vary among international professional organizations. According to the American Diabetes Association, prediabetes is defined by IFG (5.6 mmol/L ≤ fasting plasma glucose [FPG] < 7.0 mmol/L), IGT (7.8 mmol/L ≤ 2-hour postprandial glucose [2hPG] < 11.1 mmol/L), and/or glycated hemoglobin (HbA1c) levels between 5.7% and 6.5% (25). Although HbA1c is a widely utilized screening tool for glucose monitoring, its correlation with IFG and IGT remains weak (26–28). The HbA1c test remains the most commonly employed screening method for monitoring blood glucose levels, yet it does not adequately assess blood glucose variability. Therefore, it may obscure glycemic excursions, potentially underestimating the risk of both acute and chronic complications associated with prediabetes (29–31). The HbA1c measurement may also fail to capture important clinical events, such as episodes of hypoglycemia or postprandial hyperglycemia, which are crucial for assessing glycemic control (32). The diagnostic accuracy of HbA1c can be influenced by biological variability, particularly due to individual differences in red blood cell lifespan (33–36). Certain medical conditions, such as hemoglobinopathies, anemia, HIV, glucose-6-phosphate dehydrogenase deficiency, hemodialysis, recent blood transfusions, or pregnancy, may further compromise the precision of HbA1c measurements (37). It is worth noting that certain racial groups are more susceptible to conditions that can affect the accuracy of HbA1c measurements. For instance, the African American population is approximately 5.2 times more likely to have anemia than the white population, which may lead to an underestimation of HbA1c levels, thereby affecting the accuracy of its use in this group (38).

Both IFG and IGT reflect distinct pathological abnormalities in glucose metabolism. Elevated postprandial glucose levels or oral glucose tolerance test (OGTT) results are often the earliest indicators of impaired glycemic control. Insulin clamp tests have confirmed a progressive decline in islet β-cell function, even when glucose levels remain below the diagnostic threshold for prediabetes (39). Additionally, studies show that young males with fasting glucose levels below 5.6 mmol/L may still progress to T2D within six years (40). In light of this, some studies propose using a 1-hour OGTT glucose level ≥ 8.6 mmol/L as an early diagnostic marker for prediabetes, which could facilitate timely lifestyle interventions and offer significant clinical benefits (41). However, the routine use of OGTT is limited by its fasting requirement, time-consuming procedure, and the risk of hypoglycemia, which hinder its widespread adoption (42). Furthermore, the need for multiple blood draws and patient compliance issues further restrict its practicality (43). By the time hyperglycemia is detected using standard diagnostic methods, most islet β cells have already undergone irreversible damage. Therefore, identifying new biomarkers for the early detection of prediabetes is essential to improving outcomes through timely intervention.

## 4 Biomarkers discovery utilizing multi-omics techniques

**Figure 1:**
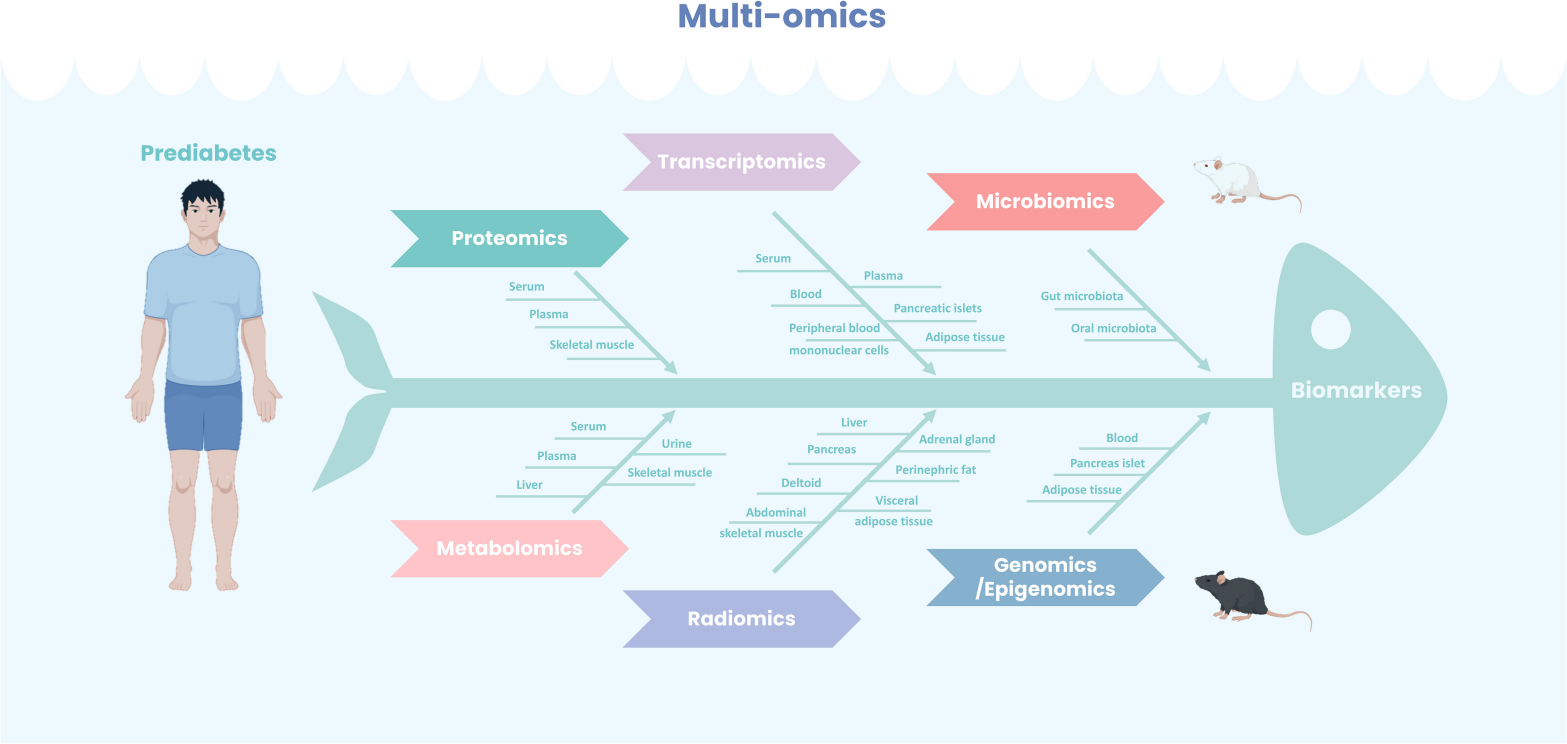
Multi-omics for prediabetes biomarkers identification. Leveraging diverse tissue sources, a comprehensive application of multi-omics methodologies—including both experimental animal models and human studies—has led to the identification of numerous potential biomarkers for prediabetes, thereby enhancing diagnostic predictive capabilities and providing a foundation for precision therapy.

Multi-omics technologies have become powerful tools for identifying biomarkers and therapeutic targets in prediabetes research. This paper provides a comprehensive review of biomarkers discovered through genomics, epigenomics, transcriptomics, metabolomics, proteomics, microbiomics, and radiomics, along with insights into future research directions (**Figure 1**).

### 4.1 Proteomics

Proteomics is increasingly recognized as a powerful approach for directly identifying biomarkers relevant to disease diagnosis. Protein biomarkers demonstrate high sensitivity and specificity in detecting T2D, providing valuable insights into both systemic and dynamic disease progression (44). Furthermore, unlike genes, proteins are subject to stringent regulation in response to cellular stimulation (45). The integration of protein biomarkers into clinical practice holds the potential to significantly improve prediabetes screening and management, ultimately enhancing patient outcomes and quality of life.

#### 4.1.1 Proteomics-based biomarkers

Liquid chromatography (LC) combined with mass spectrometry (MS) provides a high-throughput platform for large-scale protein analysis, enabling comprehensive investigation of protein expression, post-translational modifications, and interactions. The isobaric tags for relative and absolute quantitation (iTRAQ) method allows isotopic labeling and the simultaneous quantification of protein abundance from various sources. Hence, the iTRAQ-LC-MS/MS method is widely used in quantitative proteomics because of its efficiency in saving time and minimizing the number of experimental procedures when compared to traditional proteomic methods (46). Using this technique, researchers identified LAMA2, MLL4, and PLXDC2 as novel serum biomarkers for prediabetes, with 0-20% higher specificity and 20-40% greater sensitivity than FBG and HbA1c (47). MLL4 plays a key role in transcriptional activation, regulating islet β-cell function (48). LAMA2 deficiency is associated with impaired skeletal muscle metabolism, where muscle IR is a major driver of prediabetes (49). These proteins were absent or low in healthy individuals but significantly elevated in prediabetic subjects. Their combined use shows promise for developing a precise diagnostic tool for prediabetes, though further studies are needed to validate their clinical utility across diverse populations.

Selected Reaction Monitoring (SRM) is a key targeted proteomics technique for detecting and quantifying multiple proteins with high precision and specificity (50). Nano-LC/MS in SRM mode (SRM-MS) identified positive correlations between MASP1, THBS1, GPLD1, and prediabetes, while ApoA-IV showed a negative association. Bonferroni correction confirmed the positive association between MASP1, prediabetes, and fasting glucose levels. This link is likely mediated by elevated IR (51). Evidence suggests that THBS1 contributes to adipose tissue inflammation and metabolic dysregulation, explaining its elevated levels in prediabetes. A prospective cohort study using targeted SRM-MS found an independent association between MASP levels and the development of T2D and prediabetes, even after adjusting for known risk factors. Individuals with normal glucose levels who progressed to diabetes or prediabetes within 6.5 years showed significantly higher MASP levels, underscoring its predictive value (52). These proteins show promise as biomarkers, but further large-scale validation studies are essential. A label-free quantitative SRM-LC/MS/MS study revealed that when FPG ≥ 5.6 mmol/L, CD14 expression increased while CSF-1R decreased, suggesting early chronic inflammation (53). This indicates that CD14 and CSF-1R could be crucial biomarkers for prediabetes and potential new targets for therapy.

Two-dimensional electrophoresis (2-DE) combined with MS is a widely used technique to quantify disease-related protein alterations. This technique enables the simultaneous separation of hundreds to thousands of proteins from complex biological samples in a single run (54). A serum proteomics study using this method identified seven distinctive proteins: AACT, AAT, ApoA-I, HP, RBP4, TTR, and ZAG. These proteins show significant differences between individuals with normal glucose levels and those with prediabetes or diabetes (55). Most of these proteins play crucial roles in the transport, localization, and regulation of serum lipoprotein particles. Assessing the levels of these biomarkers may aid in the early diagnosis of prediabetes and diabetes.

2-DE is often regarded as an outdated technique, primarily due to its limitations in analytical depth, sample consumption, and time efficiency. As a derivative of 2-DE, two-dimensional differential gel electrophoresis (2D-DIGE) offers enhanced sensitivity, a wider linear range, more accurate quantification, and the ability to effectively separate and analyze protein isoforms, making it a more advanced alternative (56). Takahashi et al. performed a serum quantitative proteomics analysis in a prediabetic rat model utilizing 2D-DIGE and LC-multiple reaction monitoring (MRM). They successfully identified five differentially expressed proteins in prediabetes, namely A1I3, ApoE, MUG1, CRP, and SERPINA3N (57). The increased expression of SERPINA3N may lead to impaired insulin secretion in the pancreas by inhibiting Wnt/β-catenin signaling (58). These biomarkers may play a pivotal role in prediabetes pathogenesis and hold potential as diagnostic and therapeutic targets, though further replication and functional studies are needed to clarify their mechanisms.

Skeletal muscle IR is an early and critical abnormality in the development of T2D and prediabetes, making skeletal muscle a promising source for identifying biomarkers and therapeutic targets (59). Sequential window acquisition of all theoretical (SWATH) MS is an emerging MS technique that offers high quantitative accuracy, extensive proteomic coverage, reproducibility in proteome coverage, and sample throughput while minimizing the number of missing values. The application of this technology in proteomics studies has unveiled a notable decrease in proteins linked to mitochondrial energy metabolism and oxidative phosphorylation in the muscle tissues of IFG and IGT. The expression of SUPV3L1 protein is uniquely reduced in IFG muscles compared to NGT, while the expression of SNRPN is uniquely down-regulated in IGT muscles (60). These findings indicate that SUPV3L1 and SNRPN in skeletal muscle could serve as potential biomarkers for prediabetes, offering valuable insights into the pathogenesis of prediabetes from a skeletal muscle perspective. The biomarkers identified through proteomic analysis are presented in **Table 1**.

**Table 1:** Proteomics biomarkers associated with prediabetes.

Source	Species	Protein	Potential pathogenesis	Refs.
Serum	Human	LAMA2↑, MLL4↑, PLXDC2↑	Skeletal muscle metabolism, regulation of islet β cell function	(47)
Plasma	Human	MASP1↑, THBS1↑, GPLD1↑, ApoA-IV↓	IR, inflammation	(51)
Plasma	Human	MASP↑	Impaired secretory function of islet β cells	(52)
Serum	Human	CD14↑, CSF-1R↓	The early initiation of chronic inflammation	(53)
Serum	Human	AACT↓, AAT↑, ApoA-I↑, HP↑, RBP4↑, TTR↓, ZAG↑	Regulation of lipolysis, IR	(55)
Serum	Long-Evans Agouti rat	A1I3↓, ApoE↑, MUG1↑, CRP↑, SERPINA3N↑	Impaired secretory function of islet β cells	(57)
Skeletal muscle	Human	SUPV3L1↓, SNRPN↓	Dysregulation of mitochondrial RNA metabolism, abnormalities in mRNA metabolism	(60)

LAMA2, laminin subunit alpha 2; MLL4, mixed-lineage leukemia 4; PLXDC2, plexin domain containing 2; MASP, mannan-binding lectin serine peptidase; THBS1, thrombospondin 1; GPLD1, glycosylphosphatidylinositol specific phospholipase D1; ApoA, apolipoprotein A; CD14, cluster of differentiation 14; CSF-1R, colony-stimulating factor 1 receptor; AACT, alpha-1-antichymotrypsin; AAT, alpha-1-antitrypsin; HP, haptoglobin; RBP4, retinol-binding protein 4; TTR, transthyretin; ZAG, zinc-alpha2-glycoprotein; A1I3, α‐1‐inhibitor 3; MUG1, murinoglobulin‐1; ApoE, apolipoprotein E, CRP, c-reactive protein; SERPINA3N, serine protease inhibitor A3N; SUPV3L1, supervisor of mitochondrial RNA processing 3 like 1; SNRPN, small nuclear ribonucleoprotein-associated protein N.“↑” The symbol indicates upregulation of the marker in prediabetes. “↓” The symbol indicates downregulation of the marker in prediabetes.

Overall, insulin resistance and β-cell function regulation are the most common pathways affected by prediabetes biomarkers identified through proteomics. In the biological pathway of insulin resistance, the elevated levels of MASP1, THBS1, GPLD1, AAT, HP, RBP4, ZAG, and ApoA-I may be positively correlated with each other, synergistically exacerbating insulin resistance (51, 55). These biomarkers are closely associated with metabolic disturbances and may collectively influence insulin sensitivity. In contrast, the decreased levels of ApoA-IV, AACT, and TTR may also exhibit positive correlations, suggesting their potential role in alleviating insulin resistance through metabolic regulation (51, 55). On the other hand, in the pathway of β-cell function regulation, the increased levels of LAMA2, MLL4, PLXDC2, MASP, ApoE, MUG1, CRP, and SERPINA3N may act synergistically, contributing to β-cell damage and reflecting alterations in the pathway (47, 52, 57). Meanwhile, the reduced levels of A1I3 are negatively correlated with the elevated levels of other biomarkers, suggesting a protective role for A1I3 in β-cell function regulation (57).

#### 4.1.2 Limitations and future directions

Proteomic methods have identified several promising biomarkers for prediabetes, yet key challenges remain. The molecular mechanisms underlying these biomarkers remain unclear, and their sensitivity and specificity require further clarification (53). Clear concentration thresholds for diagnosing prediabetes are lacking, representing a critical area for future research (52). Many studies have relied on cross-sectional designs, limiting their ability to establish causation. Consequently, cohort studies may serve as an important approach to establishing causal relationships. Exploring biomarkers in other tissues, such as urine and saliva, offers promising avenues for future research.

### 4.2 Metabolomics

Metabolomics has become an essential high-throughput tool for exploring disease mechanisms and identifying biomarkers. Monitoring changes in metabolite levels between patients and healthy individuals provides key insights into metabolic profiles under different conditions. Targeted and non-targeted metabolomics offer complementary insights into metabolic shifts from NGT to T2D, shedding light on disease progression (61). Recent studies in metabolomics have identified promising biomarkers for diagnosing prediabetes, highlighting their potential clinical value (**Table 2**).

**Table 2:** Metabolomics biomarkers associated with prediabetes.

Source	Species	Metabolites	Potential pathogenesis	Refs.
Plasma	Spontaneously diabetic Torii	Tryptophan↓, kynurenine↓	Tryptophan metabolism	(62)
Plasma	Human	α-HB, L-GPC, oleic acid, 3-MOB, 4-MOP, α-KB	IR, decreased BCKD activity	(63)
Serum	Human	Glycine↓, lysophosphatidylcholine (LPC) (18:2) ↓, acetylcarnitine C2↑	IR, increased cellular lipid metabolites and impaired mitochondrial β-oxidation, Metabolism of arachidonic acid	(64)
Plasma	Human	Mannose↑, adrenate↑, arachidonate↑, BCAAs↑(valine, isoleucine, leucine), BCKAs↑(3-methyl-2-oxovalerate, 4-methyl-2-oxopentanoate, 3-methyl-2-oxobutyrate)	Breakdown of BCAAs, mitochondrial dysfunction	(65)
Plasma	Human	BCKAs↑, acylcarnitines↑, lysophospholipids↑, phosphatidylcholines↑	IR, impaired signaling through mammalian target of rapamycin uncoupling downstream signal transduction of insulin	(66)
Plasma/Liver	β-cell-specific prohibitin-2 knockout mice and leptin receptor–deficient mice	1, 5-dehydrosorbitol↓	Loss of functional β cell mass	(67)
Serum	Human	2-acetolactate↑, 2-hydroxy-2↑, 4-pentadienoate↑, L-arabinose↓, L-glutamine↓	Amino acid metabolism	(68)
Liver	Dahl salt-sensitive rats	L-lactic acid↑, L-propionic acid↑	acquired pyruvate oxidation deficiency, abnormal gluconeogenesis activation	(69)
Serum	Human	C18:1↑, Ala↑, Met↑, Val↑, PC aa C36:1↑, SM(OH) C22:2↑, Gly↓, Tyr↓, lysoPC a C18:2↓, PC ae C30:0↓, PC ae C42:1↓, SM C18:1↓	excessive IR-induced β oxidation, insulin-resistant and β-cell dysfunctions	(70)
Plasma	Human	C6DC↑, C14↑, C12↑, C14-OH↑, C14:1↑, Leu↓, C16↓, C14:2↑, Cit↓	The mammalian target of rapamycin signaling pathways, IR	(71)
Plasma	Human	Threonic acid↑, Indolelactic acid↑, Trimethylamine↑, 5-Hydroxy-L-tryptophan↑, Quinaldic acid↑, Nutriacholic acid↑, Pyroglutamic acid↑, L-Glutamic acid↑, Phenylacetaldehyde↓, Urea↓, L-Acetylcarnitine↓, 5-Hydroxylysine↓, Pregnenolone sulfate↓, 2-Phenylbutyric acid↓, Deoxycholic acid glycine conjugate↓, Vitamin D3↓, Sphinganine↓, 9-Methyluric acid↓	The Arginine Biosynthesis Pathway, The D-Glutamine and D-Glutamate Metabolism Pathway, The Glutathione Metabolism Pathway	(72)
Urine	Human	Male: Pentosidine glucuronide, Glutamyl-lysine sulfate, 5-(3’,4’-dihydroxyphenyl)-gamma-valerolactone-3’-O-glucuronide, 5-Phenylvaleric acid glucuronide, 3-Methoxy-4-hydroxyphenylethyleneglycol sulfate, Hippuric acid glucuronide, Cortisol glucuronide isomer, Tetrahydrocortisone glucuronide, Cortisol glucuronide isomer	–	(73)
		Female: Pentosidine glucuronide, Indoxyl sulfate, 5-(3’,4’-dihydroxyphenyl)-gamma-valerolactone-3’-O-glucuronide, Suberic acid, Aspartyl-threonine glucuronide, Glycyl-lysine	–	
Skeletal muscle	Human	Glutamate↑, ornithine↑, carnosine↑, sphingomyelins 41:1↓, sphingomyelins 41:2↓	IR, oxidative stress, glutathione synthesis pathway	(74)

The absence of a biomarker labeled up or down is unclear whether it is up-regulated or down-regulated in prediabetes. “–” The information has not been obtained from the primary publication or has not been found yet.“↑” The symbol indicates upregulation of the marker in prediabetes. “↓” The symbol indicates downregulation of the marker in prediabetes.

#### 4.2.1 Metabolomics-based biomarkers

Blood samples, including serum and plasma, are primary materials in metabolomics research due to their ability to reflect systemic physiological and pathological states. These samples are rich in metabolites and provide crucial indicators of disease onset, progression, and therapeutic response. Their accessibility and the maturity of analytical technologies make blood samples ideal for large-scale clinical and epidemiological research. Among carbohydrate biomarkers for prediabetes, mannose is the most prominent besides glucose (65). Prediabetes involves metabolic disruptions in carbohydrates, amino acids, and lipids, reflecting its complexity beyond glucose regulation. These metabolites are frequently integrated to construct a prognostic model, aiming to enhance the diagnostic accuracy of prediabetes. Elevated branched-chain amino acids (BCAAs) have been identified as early biomarkers, predicting T2D development up to ten years before onset (75, 76). Increased levels of BCAAs, including valine, leucine, and isoleucine, are frequently observed in prediabetes compared to individuals with normal glucose levels. This indicates their potential as important biomarkers for prediabetes and IR (65). Reduced tryptophan levels in prediabetes suggest its potential as a biomarker and highlight tryptophan metabolism as a promising therapeutic target (62). Other amino acids, such as glycine, glutamine, and alanine, also show distinct variations between prediabetic and normoglycemic individuals (64, 68, 70). These amino acids also possess the potential to serve as biomarkers for identifying prediabetes. Lipid metabolites, including lysophospholipids, acylcarnitine, and lysophosphatidylcholine, may serve as predictive indicators of prediabetes and T2D (64, 66). IR and mitochondrial dysfunction are key mechanisms driving these metabolic changes (61, 64–66). In summary, blood metabolites offer broad potential as biomarkers for prediabetes, and their integration into predictive models may provide novel strategies for prevention and diagnosis.

Most identified metabolites are associated with IR rather than direct changes in β-cell function. Identifying reliable biomarkers that reflect β-cell function is essential for detecting prediabetes. Using a combination of untargeted and targeted metabolomics, Li et al. employed both untargeted and targeted metabolomics to analyze the liver and plasma metabolome in two mouse models. They identified deoxyhexose 1,5-dehydrosorbitol as a biomarker reflecting the gradual loss of functional β-cell mass in asymptomatic prediabetes (67).

The liver is essential for regulating glucose homeostasis. In normal physiology, the liver regulates glycogenesis, glycogenolysis, glycolysis, gluconeogenesis, and lipogenesis in response to insulin during fasting and feeding (77). In pathological conditions, impaired hepatic insulin signaling, such as IR, disrupts metabolism, leading to hyperglycemia, inflammation, and adipose remodeling (78–80). Dahl salt-sensitive (SS) rats, which mimic prediabetic lesions, show impaired tricarboxylic acid (TCA) cycle function, reducing glucose utilization compared to salt-tolerant rats (81). Metabolomic analysis revealed higher levels of L-lactic acid and L-propionic acid in the livers of SS rats compared to salt-resistant SS.13BN rats. These findings suggest that L-lactic acid and L-propionic acid are potential hepatic biomarkers for prediabetes. Increased L-lactic acid may reflect lactic acid buildup due to impaired pyruvate oxidation, while elevated L-propionic acid suggests abnormal gluconeogenesis activation (69).

Urine offers a non-invasive and easily accessible sample material for metabolomics analysis. Despite these advantages, urine has been underutilized for identifying metabolic disease biomarkers in mass spectrometry-based metabolomics studies (82–84). Recent research developed sex-specific diagnostic models using urine metabolomics, incorporating a wide range of metabolic biomarkers. This model outperforms current blood-based parameters for IGT screening, highlighting the potential of sex-specific diagnostic models for urine-based prediabetes screening (73).

Skeletal muscle, one of the most insulin-sensitive tissues, is the primary site for insulin-stimulated glucose uptake (85). Along with the liver, skeletal muscle plays a key role in regulating glucose uptake and maintaining glucose homeostasis (86, 87). Szczerbinski et al. compared skeletal muscle metabolomics profiles in patients with varying glucose levels, revealing significant differences in glutamate, ornithine, carnosine, and sphingomyelins (41:1, 41:2) between normoglycemic and prediabetic individuals. These findings suggest these metabolites may serve as biomarkers for prediabetes and help prevent progression to T2D (74).

Insulin resistance is also the most frequently identified biological pathway in metabolomic biomarkers associated with prediabetes. Elevated levels of α-HB, L-GPC, oleic acid, 3-MOB, 4-MOP, α-KB, acetylcarnitine C2, BCKAs, acylcarnitines, lysophospholipids, phosphatidylcholines, C6DC, C14, C12, C14-OH, C14:1, C14:2, glutamate, ornithine, and carnosine may exhibit positive correlations within the biological pathways associated with insulin resistance (63, 64, 66, 71, 74). These biomarkers potentially act through multiple mechanisms, such as influencing BCKD activity, promoting the accumulation of lipid metabolites, impairing mitochondrial β-oxidation, and disrupting insulin signaling pathways, thereby exacerbating insulin resistance. Conversely, the downregulation of glycine, lysophosphatidylcholine (LPC) (18:2), leucine, C16, citrate, and sphingomyelins (41:1, 41:2) may also show positive correlations, suggesting their potential protective roles in maintaining metabolic homeostasis, which could contribute to the mitigation of insulin resistance (64, 71, 74).

#### 4.2.2 Limitations and future directions

Current metabolomics studies have identified distinct metabolic profiles across various samples, including blood, urine, liver, and skeletal muscle, demonstrating strong predictive potential for prediabetes risk. However, as with proteomics, most studies are cross-sectional, identifying metabolic differences between prediabetic and normoglycemic groups without validation through cohort studies. Limited sample sizes in current studies hinder the exploration of correlations and replication. The mechanisms underlying differential metabolites remain speculative and require further validation. Current research relies heavily on animal models, requiring clinical validation to assess diagnostic efficacy and therapeutic potential (69). Saliva, as a non-invasive, stable, and readily accessible sample, shows promise as a superior alternative to other bodily fluids for biomarker identification (88, 89). Saliva metabolomics holds significant potential for identifying biomarkers, presenting new opportunities for scientific research.

### 4.3 Transcriptomics

Transcriptomics studies RNA transcripts in specific cells or tissues, using techniques such as bulk RNA sequencing and single-cell RNA sequencing (scRNA-seq), often integrated with polymerase chain reaction (PCR) technologies (90). Gene expression can be analyzed at both bulk and single-cell levels, with scRNA-seq providing high-throughput insights into individual cell behavior. This approach provides a comprehensive view of gene expression, revealing differences among cells of various types, states, and functions. Identifying differentially expressed genes and mapping them to biological pathways deepens our understanding of genetic regulation networks (91). Transcriptomics facilitates the discovery of disease biomarkers, improving diagnostic and predictive accuracy.

#### 4.3.1 Transcriptomics-based biomarkers

Most transcriptomic studies on prediabetes utilize blood samples due to their accessibility, minimal invasiveness, and reproducibility. A recent study identified four mRNAs—ZBP1, DDX58, NFKB1, and CHUK—with elevated expression in prediabetic individuals compared to healthy controls or T2D patients. These mRNAs are involved in the cGAS/STING and NOD-like receptor (NLR) pathways, which are critical in inflammation-driven IR (92). Lee et al. identified differential mRNA expression of inflammation and lipogenesis genes—such as FCGR2b, LGALS1, VCAM1, IGFBP5, and GAS6—in a high-fat diet-induced prediabetic mouse model (93). Microarray analysis revealed distinct mRNA expression profiles in prediabetic patients compared to normoglycemic controls. These mRNAs are involved in key biochemical pathways, including starch and sucrose metabolism, pantothenate and coenzyme A biosynthesis, and niacin metabolism (94).

The transcriptomic landscape of prediabetes extends beyond mRNA, with non-coding RNAs (ncRNAs) playing key roles. Long ncRNAs (lncRNAs), over 200 nucleotides in length, lack protein-coding capacity but regulate gene expression via epigenetic, transcriptional, and post-transcriptional processes (95, 96). Dysregulated lncRNAs have been implicated in various diseases, making them promising biomarkers and therapeutic targets (97). LncRNAs influence IR by regulating lipid, carbohydrate metabolism, and inflammation (98). Several lncRNAs identified via microarray and validated internally play crucial roles in the pathophysiology of prediabetes (94). LncRNA H19 regulates lipid, glucose, and immune metabolism, showing high sensitivity for identifying prediabetes (99). It promotes hyperglycemia by upregulating hepatic FoxO1 expression and enhancing gluconeogenesis (100). Additionally, lncRNA H19 shows diagnostic and predictive potential for diabetes-related microvascular complications (99). LncRNA HCG27_201, with 91% sensitivity and 64% specificity, is a promising biomarker for prediabetes, though its mechanism requires further study (101).

MicroRNAs (miRNAs), 19 to 22 nucleotides long, are small non-coding RNAs that regulate post-transcriptional gene expression (102). MiRNAs regulate key processes such as proliferation, differentiation, and apoptosis by modulating protein translation (103). Prediabetes involves hyperglycemia-induced stress, with miRNAs playing a key role in regulating this response (104). Changes in serum or plasma miRNA profiles are commonly observed in individuals with prediabetes (105). MiRNAs regulate glucose homeostasis, insulin production, and secretion (106, 107). MiR-126-3p outperforms HbA1c in detecting prediabetes, offering greater diagnostic precision (108). TaqMAN-based RT-qPCR identified elevated miR-375 and miR-9, two islet-specific miRNAs, in prediabetic patients (109). They play a crucial role in glucose homeostasis and the pathogenesis of diabetes by regulating insulin secretion mechanisms (110). While most prediabetes biomarkers are also present in diabetes, miR-192 and miR-193b levels are elevated exclusively in prediabetes, with no increase in T2D. This differential expression suggests that prediabetes reflects a dynamic adaptation to metabolic changes, potentially progressing to diabetes (111). Most studies have focused on male or mixed-sex cohorts, lacking female-specific miRNA profiles for prediabetes. Kovac et al. divided female patients into IFG and NGT groups, profiling plasma miRNAs and validating them in islet cells and adipocytes of female mice. They found elevated let-7i-5p levels under prediabetic conditions. Let-7i-5p may regulate systemic insulin sensitivity and serve as a biomarker for prediabetes in women by impairing insulin signaling and glucose homeostasis (112).

Circular RNAs (circRNAs) are closed-loop non-coding RNAs involved in key biological processes across eukaryotes and prokaryotes (113, 114). CircRNAs act as miRNA sponges, sequestering miRNAs and regulating their target genes by competing for miRNA binding sites (115–117). CircRNAs such as hsa_circ_0063425, hsa_circ_0056891, hsa_circ_0111707, and hsa_circ_0071336 regulate target gene expression by sequestering miRNAs, highlighting their potential as prediabetes biomarkers (118–120). **Table 3** delineates the potential biomarkers identified through transcriptomic methodologies.

**Table 3:** Transcriptomic biomarkers associated with prediabetes.

Source	RNA classification	Species	RNA transcript	Potential pathogenesis	Refs.
Serum	mRNA	Human	ZBP1↑, DDX58↑, NFKB1↑, CHUK↑	cGAS/STING, NOD-like Receptor Pathways	(92)
Serum	mRNA	High-fat diet-induced pre-DM mouse model	FCGR2b↑, LGALS1↑, VCAM1↑, IGFBP5↑, GAS6↑	Inflammation and lipogenesis	(93)
Blood	mRNA	Human	BX571672.2↑, RP11-195E2.4↑, SIRPB2↓, HIST1H4D↓, CCNJL↓, CADM1↑, KCNJ2↓, CASP5↓, IL1B↓, MGAM↓	Starch and sucrose metabolism, pantothenate and coenzyme A biosynthesis, and nicotinate and nicotinamide metabolism	(94)
Serum	lncRNA	Human	lncRNA HCG27_201↓	–	(101)
Plasma	lncRNA	Human	lncRNA H19↓	–	(99)
Whole Blood	lncRNA	Human	lncRNA ENST00000550337.1↑	**-**	(121)
Blood	lncRNA	Human	NONHSAT010921↑, NONHSAT108315↑, lnc-SOX6-6↓, NONHSAT070281↓, NONHSAT122651↓, NONHSAT081137↑, NONHSAT073729↓, NONHSAT123762↓, NONHSAT058496↑, NONHSAT096326↑	**-**	(94)
Plasma	miRNA	Human	has-miR-1249↓, has-miR-320b↓, has-miR-572↑	**-**	(103)
Whole Blood	miRNA	Human	miR-1299↑, miR-126-3p↑, miR-30e-3p↑	**-**	(108)
Serum	miRNA	Human	miR-192↑, miR-193b↑	**-**	(111)
Plasma	miRNA	Human	let-7b↑, miR-142↓, miR-144↑, miR-29a↑	IR	(122)
Plasma	miRNA	Human	miR-21↑	ROS damage	(123)
Plasma	miRNA	Human	miR-145-5p↓	–	(124)
Plasma/Pancreatic islets/white adipose tissue	miRNA	Human/Mouse (female)	let-7i-5p↑	Impairment of insulin signaling in pancreatic β cells	(112)
Plasma	miRNA	Human	miR-148b-3p↑, miR-27a-3p↓	IR	(125)
Whole Blood	miRNA	Human	miR-30a-5p↑, miR-182-5p↑	Pancreatic β cell dysfunction	(126)
Whole Blood	miRNA	Human	miR-375↑, miR-9↑	Pancreatic β cell dysfunction	(109)
Peripheral blood mononuclear cells	circRNA	Human	hsa_circ_0111707↓	–	(118)
Peripheral blood mononuclear cells	circRNA	Human	hsa_circ_0063425↓, hsa_circ_0056891↓	PI3K/AKT signaling pathway	(119)
Peripheral blood mononuclear cells	circRNA	Human	hsa_circ_0071336↓	**-**	(111)

ZBP1, Z-DNA-binding protein 1; DDX58, DexD/H-Box Helicase 58; NFKB1, Nuclear Factor Kappa B Subunit 1; CHUK, conserved helix–loop–helix ubiquitous kinase; FCGR2b, fc receptor, IgG, low affinity Iib; LGALS1, lectin, galactose binding, soluble 1; VCAM1, vascular cell adhesion molecule 1; IGFBP5, insulin-like growth factor binding protein 5; GAS6, growth arrest specific 6; SIRPB2, signal regulatory protein β 2; HIST1H4D, histone cluster 1 H4 family member d; CCNJL, cyclin J-like; CADM1, cell adhesion molecule 1; KCNJ2:Potassium inwardly rectifying channel subfamily J member 2; CASP5, caspase 5; IL1B, Interleukin 1 β; MGAM, Maltase-glucoamylase. “–” The information has not been obtained from the primary publication or has not been found yet.“↑” The symbol indicates upregulation of the marker in prediabetes. “↓” The symbol indicates downregulation of the marker in prediabetes.

Insulin resistance and dysregulation of pancreatic β-cell function are the predominant pathways implicated in transcriptomics-based biomarkers of prediabetes. In the biological pathways underlying insulin resistance, the upregulation of let-7b, miR-144, miR-29a, and miR-148b-3p may be positively correlated, collectively contributing to the exacerbation of insulin resistance through various mechanisms (122, 125). In contrast, the downregulation of miR-27a-3p and miR-142 may also be positively correlated, suggesting a protective role in modulating insulin resistance, potentially alleviating its effects. In the context of pancreatic β-cell dysfunction, the upregulation of miR-30a-5p, miR-182-5p, miR-375, and miR-9 may interact synergistically, adversely affecting β-cell function and further exacerbating β-cell damage (109, 126). These changes in miRNA expression highlight their involvement in the complex regulatory networks associated with both insulin resistance and β-cell dysfunction.

#### 4.3.2 Limitations and future directions

The current research is limited by the lack of large-sample validation, multi-population studies, and prospective trials to confirm these biomarkers’ role in diabetes risk screening strategies. Many mechanisms remain unclear, requiring further experimental studies to explore the regulatory roles of ncRNAs in downstream gene expression and their entry into the bloodstream (118, 124). Differential expression of prediabetic RNAs is mainly observed in serum and plasma, indicating potential for future exploration in diverse tissues.

### 4.4 Genomics

Genomics focuses on analyzing genome sequences and linking them to molecular and phenotypic traits (127). It explores genetic variations such as single nucleotide polymorphisms (SNPs) and chromosomal abnormalities, which are associated with disease susceptibility. Such variations influence health outcomes and treatment responses. Genomics employs tools such as genotype microarrays, next-generation sequencing, and exome sequencing. The field has experienced a surge in available genomic data (128). Genome-wide association studies (GWAS) identify genetic variants associated with complex diseases and traits by analyzing large sample sizes. Through GWAS, researchers have uncovered significant associations between specific genotypes and increased disease risk.

#### 4.4.1 Genomics-based biomarkers

**Figure 2:**
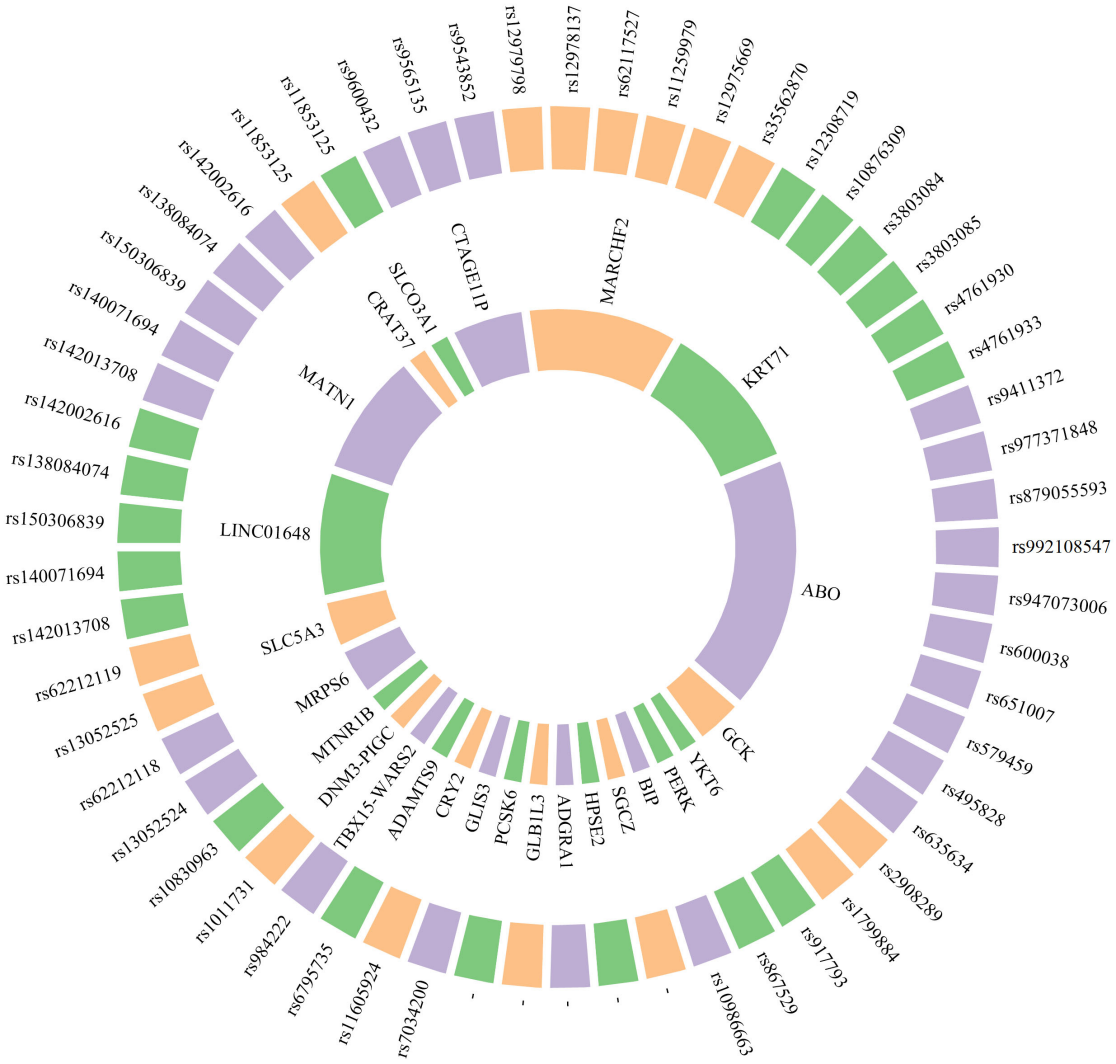
Genomics biomarkers associated with prediabetes. The inner circle denotes the gene name, while the outer circle illustrates the corresponding SNP associated with that gene. “–” The information has not been obtained from the primary publication or has not been found yet.

Genomic approaches have elucidated numerous biomarkers of considerable significance associated with prediabetes (**Table 4**, **Figure 2**). Feng et al.’s study indicated that allele C of SNP rs867529 at the PERK gene locus is associated with an increased risk of prediabetes, whereas allele G of SNP rs10986663 in the BIP gene is inversely correlated with prediabetes risk. Genetic variations in these genes may play a role in the development of endoplasmic reticulum stress (130). A GWAS-based on changes in prediabetes status has identified five novel genes associated with changes in prediabetes status, including SGCZ at 8p22, HPSE2 at 10q24.2, ADGRA1 at 10q26.3, GLB1L3 at 11q25, and PCSK6 at 15q26.3 (131). Sparsø et al. discovered that the G allele of MTNR1B rs10830963 is associated with an increased risk of isolated IFG in a large sample of European populations (134).

**Table 4:** Genomics biomarkers associated with prediabetes.

Species	Genes	Potential pathogenesis	Refs.
Human	CTAGE11P, MARCHF2, KRT71, ABO	IR and abnormal insulin secretion	(129)
Human	PERK, BIP	endoplasmic reticulum stress	(130)
Human	SGCZ, HPSE2, ADGRA1, GLB1L3, PCSK6	–	(131)
Human	GLIS3, CRY2	Impaired islet β cell function	(132)
Human	ADAMTS9, TBX15-WARS2, DNM3-PIGC	Central obesity	(133)
Human	MTNR1B	Pancreatic β cell dysfunction	(134)
Human	MRPS6, SLC5A3, LINC01648, MATN1, CRAT37, SLCO3A1	–	(135)
Human	GCK, YKT6	Initiation of abnormal glucose homeostasis, development of microalbuminuria	(136)

CTAGE11P, cutaneous T cell lymphoma-associated antigen 11 pseudogene; MARCHF2, membrane-associated ring-CH-type finger 2; KRT71, keratin 71; ABO, ABO blood group system; PERK, protein kinase RNA-like endoplasmic reticulum kinase; BIP, binding immunoglobulin protein; SGCZ, sarcoglycan zeta; HPSE2, heparanase 2; ADGRA1, adhesion G protein-coupled receptor A1; GLB1L3, galactosidase beta 1 like 3; PCSK6, proprotein convertase subtilisin/kexin type 6; GLIS3, GLIS family zinc finger 3; CRY2, cryptochrome circadian regulator 2; ADAMTS9, a disintegrin and metalloproteinase with thrombospondin motifs 9; TBX15-WARS2, TBX15-WARS2 fusion gene; DNM3-PIGC, DNM3-PIGC fusion gene; MTNR1B, melatonin receptor 1B; MRPS6, mitochondrial ribosomal protein S6; SLC5A3, solute carrier family 5 member 3; LINC01648, long intergenic non-protein coding RNA 1648; MATN1, matrilin 1; CRAT37, carnitine acetyltransferase 37; SLCO3A1, solute carrier organic anion transporter family member 3A1; GCK, glucokinase; YKT6, ykt6 v-SNARE homolog. “–” The information has not been obtained from the primary publication or has not been found yet.

The correlation between genetic susceptibility markers and the prediabetes phenotype has been a prominent focus of research in recent years. Lin et al. utilized GWAS technology to investigate the Chinese Han population, revealing a significant association between genetic variations of CTAGE11P, MARCHF2, KRT71, ABO and other genes with fasting proinsulin (FPI), fasting insulin (FI), 2 hours postprandial proinsulin (2hPI) and 2 hours postprandial insulin (2hI). These genetic variants may be indicative of IR and abnormal insulin secretion, holding promise as potential biomarkers for prediabetes prevention (129). The MRPS6/SLC5A3 gene rs13052524 and rs62212118 sites were found to be correlated with 2hPG levels in prediabetic patients from Hainan, China, according to a study conducted there. Six loci within four prediabetic genes (LINC01648, MATN1, CRAT37, and SLCO3A1) exhibited associations with HbA1C. This indicates that these loci may play a role in the regulation of 2hPG and HbA1C levels (135). Genetic loci associated with waist-hip ratio, such as rs6795735 (ADAMTS9), rs984222 (TBX15-WARS2) and rs1011731 (DNM3-PIGC), were found to be linked to the risk of IFG, indicating a potential association between genetic predisposition to central obesity and impaired islet function (133). Wang et al. conducted a GWAS and identified two independent significant sites (rs9457733 and rs11243373) as well as 37 candidate genes associated with prediabetic 25 (OH) D3, indicating its importance as a risk factor for prediabetes (137). Choi et al. discovered that GCK and YKT6 were susceptibility loci associated with prediabetes in a GWAS study involving two population cohorts, a control group, and a prediabetic group. Additionally, polymorphisms of these genetic variants were independently linked to an elevated risk of microalbuminuria. This suggests that the genetic mechanism of prediabetes may serve as a predictor for the risk of renal microvascular complications to some extent, thereby laying the groundwork for early detection and management of high-risk patients (136).

#### 4.4.2 Limitations and future directions

Prediabetes often arises from a combination of minor variations in multiple genes and the interplay between genetic factors and environmental influences, which presents challenges in interpreting and applying genetic test results. Most studies do not stratify SNPs by age/sex to investigate age/sex-specific differences in SNP effects (130, 131, 134). Furthermore, environmental factors such as lifestyle, diet, and physical activity significantly impact the onset and progression of prediabetes (138). However, genomics struggles to comprehensively encompass the influence of these factors. Studies based on GWAS can only identify correlations, not causations. Therefore, it is essential to validate the association between genetic loci or candidate genes and prediabetes through molecular experiments. Current genomic studies are limited by relatively small sample sizes. Future large-scale genomic research will be essential to validate the identified SNPs and uncover additional genetic loci associated with prediabetes (131).

Although genomic biomarkers have been identified in European and Chinese populations, their applicability in underrepresented populations remains to be fully explored, given the genetic diversity across different ethnic and geographic groups. For example, cross-population GWAS could validate the relevance of these biomarkers in other populations and potentially identify novel genetic markers specific to these groups. Such efforts would contribute to a better understanding of the genetic heterogeneity of prediabetes across diverse populations and support the development of precision medicine and global prediabetes diagnostic strategies.

### 4.5 Epigenomics

Epigenetic modifications, such as DNA methylation and histone alterations, regulate gene expression without altering the DNA sequence and play a key role in prediabetes pathophysiology. Through epigenomic analysis, researchers can identify DNA methylation changes in gene promoters and histone modifications linked to prediabetes traits. Advanced technologies, including whole-genome methylation sequencing and ChIP-seq, enable the discovery of specific biomarkers. These findings provide essential support for early diagnosis and personalized treatments, offering new avenues for precision medicine in prediabetes management (139, 140).

#### 4.5.1 Epigenomics-based biomarkers

As a common epigenetic modification, DNA methylation involves adding methyl groups to the fifth carbon of cytosine in cytosine-phosphate-guanine (CpG) dinucleotides, catalyzed by DNA methyltransferase (141, 142). Hypermethylation in gene promoters impedes transcription factor binding, inhibiting transcription and silencing gene expression. In contrast, hypomethylation enhances transcription factor binding, often increasing gene expression. Using methylated DNA immunoprecipitation (MeDIP) technology, Matsha et al. identified distinct DNA methylation patterns in South African individuals of mixed ancestry, showing both hypermethylation and hypomethylation in gene promoters. These changes affect genes involved in immune function, signal transduction, glucose transport, and pancreatic development. The affected pathways include linoleic acid and arachidonic acid metabolism, both showing hypomethylation in prediabetes (143). This study lays a foundation for exploring epigenetic mechanisms in prediabetes and identifying potential biomarkers.

Testosterone and estrogen directly affect skeletal muscle, liver, and adipose tissue, influencing carbohydrate metabolism. They also indirectly influence metabolism by modulating body fat quantity and distribution (144, 145). These hormones thus play a pivotal role in glucose regulation and prediabetes pathogenesis. Peripheral blood DNA methylation analysis in a case-control study found a positive correlation between AR gene methylation and IFG in females. Serum testosterone negatively modulated the link between AR gene methylation and IFG, suggesting that low testosterone and AR methylation levels reduce IFG risk (146). This phenomenon may be attributed to the capacity of testosterone to ameliorate IR and dysregulated glucose metabolism resulting from AR gene methylation by modulating the expression or activity of the AR, thereby reducing the risk of IFG. Feng et al. examined how estrogen receptor α (ESRα) methylation and progesterone influence glucose metabolism disorders in a case-control study. In men and postmenopausal women, CpG 1 methylation in the ESRα gene and elevated progesterone levels were positively associated with IFG. Individuals with both high CpG 1 methylation and progesterone levels showed a higher IFG risk (147). This mechanism likely involves IR caused by ESRα methylation, progesterone levels, and pancreatic secretory cell apoptosis. These factors may act synergistically through the PI3K/AKT pathway, but further research is needed to clarify the underlying mechanisms (148–150).

In late pregnancy, hormonal fluctuations increase IR, requiring greater insulin secretion by pancreatic β cells to maintain normal blood glucose (151). If β-cell secretion fails to meet increased demand, hyperglycemia occurs. A cohort study using DNA methylation assays identified differential methylation at three CpG sites—LINC00917, TRAPPC9, and LEF1—in women with abnormal glucose tolerance. LINC00917 and TRAPPC9 sites were independently linked to glucose tolerance status four years postpartum, suggesting their role in postprandial prediabetes (152).

Modifications to histones alter their structure and function, influencing chromatin architecture and gene expression. Histone methylation is a key post-translational modification; for example, H3K4me3 refers to the trimethylation of histone H3’s fourth lysine residue. The myeloid/lymphoid or mixed-lineage leukemia (MLL) gene encodes a histone methyltransferase that methylates H3K4, modulating gene transcription (153). Yoshino et al. demonstrated the crucial role of the MLL gene in pancreatic β cells. In βHC-9 cells with MLL knockout, insulin secretion declined, along with reduced expression of glucose-sensitive genes SLC2A1 and SLC2A2. Moreover, MLL heterozygous knockout mice showed impaired glucose tolerance and reduced insulin secretion (154). These results suggest that MLL genes contribute to prediabetes pathogenesis through histone modifications. Castellano-Castillo et al. provided the first analysis of H3K4me3 markers on gene promoters involved in lipogenesis, lipid metabolism, and inflammation in human visceral adipose tissue. They found significant enrichment of H3K4me3 at the promoters of E2F1, LPL, SREBF2, SCD1, PPARG, and IL6 in lean individuals with normal glucose compared to obese individuals with prediabetes. This suggests that H3K4me3 plays a critical role in prediabetes onset and progression (155). **Table 5** presents potential biomarkers identified through epigenomic analysis.

**Table 5:** Epigenomics biomarkers associated with prediabetes.

Source	Species	Biomarkers or key pathways	Ref.
Peripheral blood	Human	Linoleic acid and arachidonic acid metabolism	(143)
Peripheral blood	Human	AR, testosterone	(146)
Peripheral blood	Human	ESRα, progesterone, PI3K/AKT signaling pathway	(147)
Peripheral blood	Human	LINC00917, TRAPPC9, LEF1	(152)
Pancreas islet	Mouse	MLL, SLC2A1, SLC2A2	(153)
Adipose tissue	Human	E2F1, LPL, SREBF2, SCD1, PPARG, IL6	(155)

LINC00917, long intergenic non-protein coding RNA 917; TRAPPC9, trafficking protein particle complex subunit 9; LEF1, lymphoid enhancer-binding factor 1; SLC2A, solute carrier family 2 member; E2F1, E2F transcription factor 1; LPL, lipoprotein lipase; SREBF2, sterol regulatory element-binding transcription factor 2; SCD1, stearoyl-CoA desaturase 1; PPARG, peroxisome proliferator-activated receptor gamma.

#### 4.5.2 Limitations and future directions

In the field of epigenomics related to prediabetes, current research has predominantly focused on 5-methylcytosine (5mC) and 5-hydroxymethylcytosine (5hmC). However, emerging epigenetic modifications, such as N6-methyladenine (6mA) and other non-canonical DNA modifications, remain underexplored in this context. Future studies should investigate these additional modifications to gain a more comprehensive understanding of the epigenomic landscape and their potential roles in prediabetes pathogenesis. MeDIP cannot distinguish between 5mC and 5hmC or identify differentially methylated CpG sites. Advanced techniques are needed for more precise DNA methylation analysis in future research. Although some studies used peripheral blood leukocytes, future research should focus on tissues more relevant to prediabetes, such as pancreatic β cells, due to variations in methylation across blood cell types (143, 146). Moreover, future studies should control for confounding factors like smoking and obesity to reduce potential interference. Since many studies are observational case-control studies, cohort studies are needed to establish causality and provide further validation (146). Animal study findings must be validated in clinical settings.

### 4.6 Microbiomics

#### 4.6.1 Gut microbiome-based biomarkers

The gut microbiome, consisting of trillions of microbes residing in the gastrointestinal tract, has been recognized as a virtual organ that interacts with the gut and other organs in the host’s body to facilitate various physiological processes (156). Multiple research has shown that gut microbiota and its metabolites are essential in controlling inflammation, maintaining energy equilibrium, and regulating lipid and glucose metabolism (157–159). Emerging evidence reveals significant gut microbiota dysbiosis in individuals with prediabetes. High-throughput 16S rRNA sequencing, widely used in microbiome research, provides insights into bacterial diversity and community structure in health and disease.

Prediabetes alters gut microbial structure, reducing both diversity and relative abundance (160). Compared to individuals with NGT, there is a notable decrease in the presence of bacteria that produce short-chain fatty acids (SCFAs) in the gut microbiota of those with prediabetes, including genera such as Anaerostipes, Enterococcus, Intestinibacter, Faecalibacterium prausnitzii, Roseburia and others (161–164). The primary SCFAs present in the intestinal tract are acetate, propionate, and butyrate. The population of butyrate-producing bacteria in the intestines of individuals with prediabetes is notably reduced, including species such as Roseburia, Faecalibacterium prausnitzii, Clostridium spp., Alistipes spp., Pseudoflavonifractor spp., Oscillibacter spp., and others (161, 163, 165, 166). In contrast, an increased abundance of some opportunistic pathogenic bacteria was observed, including species from Enterobacteriaceae, Streptococcus, Pseudomonadota, and Shigella spp./Escherichia spp., among others (162, 164, 166–168). The increased presence of opportunistic pathogens and bacteria-derived lipopolysaccharides can lead to the development of low-grade inflammation and IR (169, 170), both of which are key characteristics of prediabetes. **Table 6** summarizes the potential biomarkers for prediabetes identified through gut microbiota profiling.

**Table 6:** Microbiomics biomarkers associated with prediabetes.

Gut/oral microbiota	Species	Enrichment	Depletion	Refs.
Gut microbiota	Human	Dorea spp., Pseudomonadota, Enterobacterales, Shigella spp. & Escherichia spp., Bacilli, Lactobacillales, Streptococcaceae, Streptococcus, Eisenbergiella, Eisenbergiella massiliensis, Neglecta, Neglecta timonensis	Roseburia sp., Roseburia intestinalis, Faecalibacterium prausnitzii, Dialister invisus, Veillonella spp., Sutterella wadsworthensis, Oscillibacter valericigenes, Bacteroides coprocola	(162)
	Human	Collinsella, Allisonella, Escherichia/Shigella, Senegalimassilia, Prevotella_9, Granulicatella, Veillonella	Intestinibacter, Enterococcus, Anaerostipes, Blautia	(161)
	Human	Dorea, Ruminococcus, Sutterella, Streptococcus	Clostridium, Akkermansia muciniphila	(167)
	Human	Comamonadaceae	**-**	(171)
	Human	Megamonas, Haemophilus, norank_p_Saccharibacteria	Ruminococcaceae, Barnesiella, Sutterella, Ruminiclostridium, Clostridiales, Coriobacteriaceae, Ruminiclostridium, Flavonifractor	(160)
	Human	–	Roseburia	(163)
	Zucker Diabetic Sprague Dawley rat	Alistipes, Ruminococcus	Lactobacillus	(172)
	Human	An unknown genus from family Pseudonocardiaceae	–	(173)
	Human	Bproteobacteria, genus Prevotella, genus Megamonas, Clostridiales sp. SS3/4	Verrucomicrobia, Verrucomicrobiae, Streptococcus, Akkermansia muciniphila ATCCBAA-835, Faecalibacterium prausnitzii L2-6	(166)
	Human	Escherichia coli, Streptococcus salivarius, Eggerthella sp., Megasphaera elsdenii	Clostridia class (e.g. Dialister invisus, Roseburia hominis), Faecalibacterium prausnitzii	(164)
	Human	Blautia genus, Serratia genus	**-**	(174)
	Human	Escherichia, Veillonella	Blautia, Anaerostipes	(175)
	Human	Clostridium bolteae	Faecalibacterium spp., Clostridium spp., Alistipes spp., Pseudoflavonifractor spp., Oscillibacter spp.	(165)
	Human	Megasphaera elsdenii, Streptococcus equinus/gallolyticus/infantarius/lutetiensis, Prevotella_9, Alistipes finegoldii/onderdonkii, Mitsuokella, Escherichia/Shigella albertii/boydii/coli/dysenteriae/fergusonii/flexneri/sonnei/vulneris, Megasphaera, Prevotella_2, Vibrio cholerae, Lactobacillus, Alloprevotella, Rhodococcus baikonurensis/boritolerans/degradans/erythropolis/ globerulus/hoagii/opacus/qingshengii/rhodochrous,	Prevotella_9, Phascolarctobacterium faecium, Barnesiella intestinihominis, Flavonifractor plautii, [Family]Ruminococcaceae, Tyzzerella_4 nexilis, Bacteroides nordii, Faecalibacterium, Agathobacter, [Family]Muribaculaceae, Ruminococcaceae_UCG-002, Christensenellaceae_R-7_group, Ruminococcaceae_UCG-010	(168)
	Human	Blautia obeum, Blautia wexlerae, Clostridium clostridioforme, Ruminococcus gnavus	Bacteroides dorei, Coprococcus eutactus, Eubacterium eligens, Bacteroidetes eggerthii	(176)
Oral microbiota	Human	Absconditabacteriales, Stomatobaculum, Leptotrichia, Campylobacter	Alloprevotella, Neisseria, Rothia, Streptococcus, Ruminococcaceae, Leptotrichia	(177)

**Figure 3:**
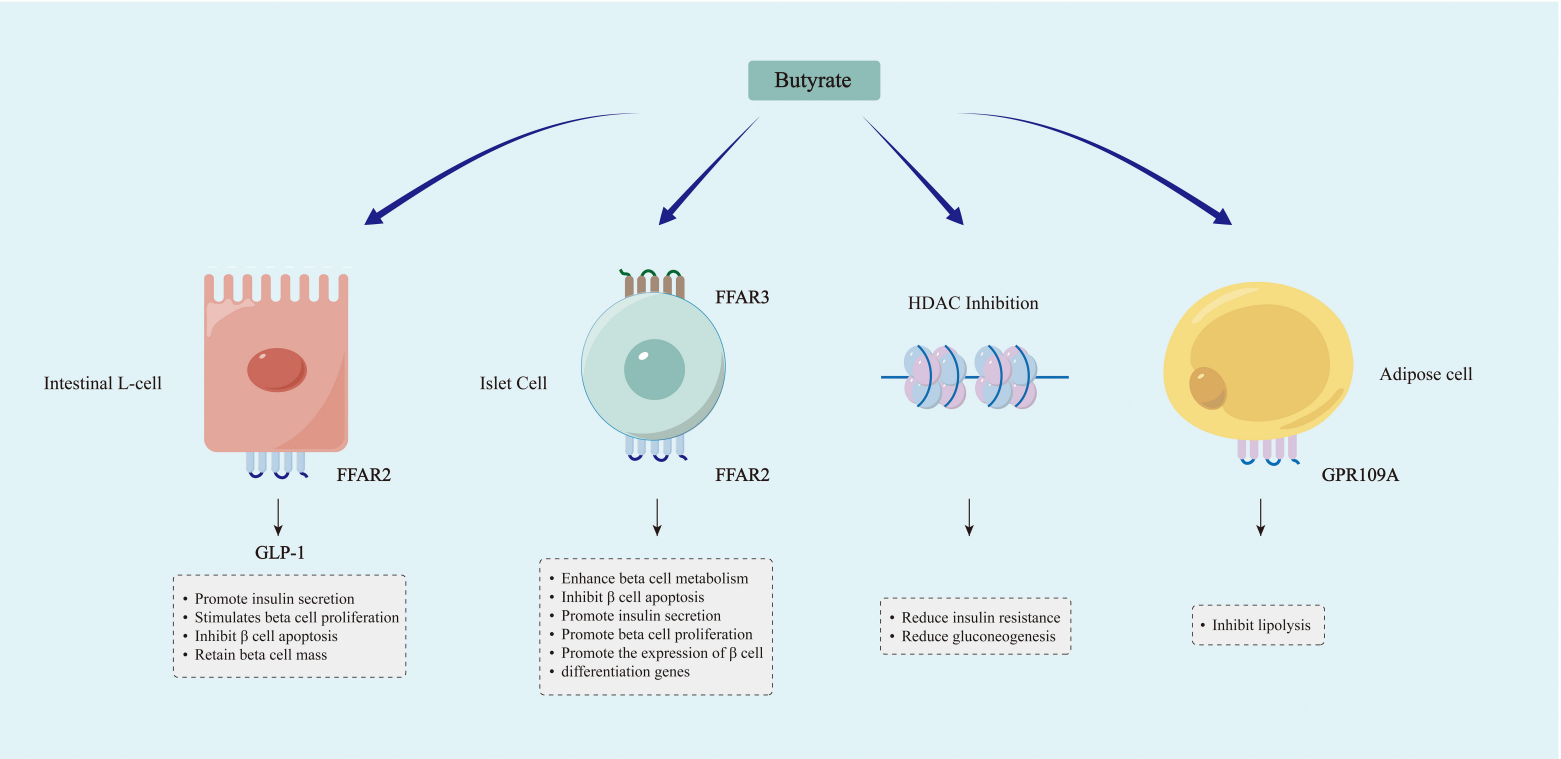
Mechanisms through which SCFAs, particularly butyrate, modulate blood glucose levels. Butyrate and other SCFAs can induce the secretion of GLP-1 by activating FFAR2 receptors on intestinal L cells, thereby modulating the metabolism of islet β cells. Butyrate and other SCFAs are involved in the metabolism of islet β cells through their binding to FFAR2 and FFAR3 receptors in islet cells. Butyrate modulates gene expression through the promotion of histone acetylation, leading to enhanced insulin sensitivity and decreased gluconeogenesis. SCFAs like butyrate may enhance glucose homeostasis by inhibiting lipolysis.

As the main product of dietary fiber fermentation in the large intestine, butyrate provides a critical energy source for colon cells (178). Butyrate and the bacteria that produce it promote anti-inflammatory properties, maintain intestinal immune homeostasis, and can also enhance insulin sensitivity and glucose tolerance (179, 180). The mechanisms through which SCFAs like butyrate regulate blood glucose may include several key aspects (**Figure 3**).

Butyrate supplementation has been demonstrated to elevate levels of glucagon-like peptide-1 (GLP-1) and significantly enhance glycemic control in individuals with T2D (181). The hormone GLP-1, produced in the intestines, acts to stimulate pancreatic β cells and induce the secretion of insulin for the regulation of glucose levels. Butyrate and other SCFAs stimulate the secretion of GLP-1 by binding to FFAR2 receptors on intestinal L cells (182). The GLP-1R (receptor) gene is also expressed in β cells, and butyrate can induce activation of the GLP-1R gene and subsequent release of GLP-1 (183). The potential mechanism involves GLP-1 promoting the upregulation of cAMP (cyclic adenosine monophosphate), leading to postprandial insulin secretion through acceleration of glucose-dependent closure of ATP-regulated potassium channels (184–186). In addition to its pro-insulin effect, GLP-1 also maintains pancreatic β cell mass by promoting β cell proliferation and inhibiting β cell apoptosis, and has been utilized in the transdifferentiation of insulin-producing cells (187–189).

The impact of SCFAs extends beyond the gastrointestinal tract, as they are absorbed into the peripheral circulation and contribute to tissue metabolism. Butyrate signaling plays a crucial role in islet function. Butyrate and other SCFAs can serve as ligands for G protein-coupled receptors (GPR), binding to free fatty acid receptor 3 (FFAR3) (GPR41) and FFAR2 (GPR43), among others. Both GPR41 and GPR43 are expressed in pancreatic islets (190, 191). Through GPR41 signaling, butyrate can enhance β cell metabolism and inhibit islet cell apoptosis (192). On the other hand, by activating GPR43, SCFAs such as butyrate can stimulate insulin secretion, β cell proliferation, and the expression of β cell differentiation genes, indicating that GPR43 holds promise as a target for future research (193).

SCFAs, primarily butyrate, function as a natural inhibitor of histone deacetylase (HDAC) and regulate gene expression through epigenetic mechanisms (194). Animal experiments have demonstrated that sodium butyrate may ameliorate IR, dyslipidemia, fat accumulation, and gluconeogenesis in rats by inducing hyperacetylation of histone H3, thereby enhancing glucose homeostasis (195). Furthermore, it has been demonstrated that sodium butyrate can enhance insulin sensitivity in skeletal muscle cells exposed to long-term palmitate by promoting hyperacetylation of insulin receptor substrate-1 (196). Therefore, the reduction of butyrate may lead to IR through epigenetic mechanisms, subsequently causing disorders in blood glucose metabolism.

SCFAs binding receptors, such as GPR109A, are also present in adipose tissue. A study demonstrated that the administration of a GPR109A agonist inhibited lipolysis to improve glucose homeostasis in T2D patients (197). Therefore, it can be hypothesized that the deficiency of SCFAs, such as butyrate, may lead to impaired blood glucose regulation by accelerating lipolysis; however, further confirmation is still required through human cohort studies.

#### 4.6.2 Oral microbiome-based biomarkers

Both salivary glucose levels and pH influence the composition of the oral microbiome. Studies show that individuals with diabetes have lower salivary pH compared to non-diabetic individuals, and salivary glucose levels are closely linked to hyperglycemia (198–200). Thus, individuals with hyperglycemia are expected to display a distinct oral microbiome composition compared to healthy individuals. Significant structural changes in the oral microbiome have been observed in individuals with diabetes (201, 202). Although the oral bacterial population is significantly smaller than that of the gut, it has the potential to disseminate to other body sites, leading to localized and systemic inflammation (203, 204). These potentially harmful pathogens may have a notable influence on the development of IR, akin to the effect of the microbiota in the gut. Thus, oral microbiota dysbiosis is not only a marker of hyperglycemia but may also drive disease progression. The findings indicate that the traits displayed by the oral microbiota could serve as potential indicators of disease status. The analysis of bacterial microbiome composition in saliva has the potential to serve as a valuable non-invasive approach for diagnosing prediabetes.

The limited research in this area has resulted in no consensus on whether oral microbial abundance and diversity increase or decrease in prediabetes. One study analyzing the salivary microbiome based on HbA1c and FBG reported that prediabetic individuals exhibit greater microbial richness and diversity compared to normoglycemic individuals (177). However, a study by Saeb et al. reached the opposite conclusion, finding a significant reduction in the biological and phylogenetic diversity of the prediabetic oral microbiota (205). Rungrueang et al. discovered that the ratio of firmicutes to bacteroides in the oral microbiota of prediabetic patients was higher compared to healthy controls, consistent with previous findings in T2D. The abundance of Rothia significantly decreased in prediabetes, irrespective of whether HbA1c or FPG was used as the classification criterion (177). The mechanism may be that nitrate reductase in Rothia metabolizes nitrate to nitrite, which can be further reduced to nitric oxide (NO) in blood and muscle. NO has been shown to have a substantial impact on the control of metabolic processes, and it has the ability to enhance insulin secretion and promote muscle glucose uptake (206). Therefore, a reduction in the abundance of oral bacteria Rothia may serve as a significant indicator for the presence of prediabetes. The potential biomarkers for prediabetes, identified through oral microbiome analysis, are outlined in **Table 6**.

#### 4.6.3 Limitations and future directions

Due to the multifactorial nature of the impact on intestinal flora, it is challenging for many studies to effectively control for potential confounding variables, which may somewhat limit the generalizability of study findings. Among the various factors influencing gut microbiome composition and diversity, diet stands out as one of the most significant. For instance, a high-carbohydrate diet has been linked to elevated levels of Prevotella, while a high-fat/protein diet is associated with a predominant presence of Bacteroides (207). By adopting a plant-based diet, you can enhance the abundance of beneficial bacteria in your gut, such as Bacteroidetes, thereby supporting gastrointestinal and overall health (208). Furthermore, the administration of hypoglycemic agents, antibiotics, probiotics, and other pharmaceuticals may influence the diversity of gut microbiota. For instance, metformin treatment elevates Firmicutes while reducing Bacteroidetes (209). The use of antibiotics often results in damage to the gut microbiota. For instance, broad-spectrum antibiotics like ampicillin disrupt the microbial community and lead to a reduction in the diversity of the gut microbiota (210). Therefore, minimizing the impact of confounding factors to the greatest extent possible may be crucial for enhancing the reliability of changes observed in gut microbiota outcomes. Since some studies were cross-sectional and focused on single-race cohorts, further longitudinal studies across diverse populations are needed.

### 4.7 Radiomics

Radiomics represents a burgeoning field within medical imaging that emphasizes the high-throughput extraction and analysis of quantitative features from various imaging modalities, including magnetic resonance imaging (MRI), computed tomography (CT), and ultrasound (US) (211). Through precise feature extraction techniques and comprehensive data analysis, radiomics thoroughly explores the vast information embedded in medical images, thereby facilitating the identification of disease-specific biomarkers (212). These biomarkers elucidate the pathophysiological state, monitor disease progression, and predict treatment response, providing a critical foundation for clinical diagnosis, therapeutic decisions, and prognostic evaluations (213, 214).

#### 4.7.1 Radiomics-based biomarkers

Visceral adipose tissue (VAT) serves as a critical predictor of IR and is prominently featured as a biomarker in imaging studies related to diabetes and prediabetes (215). The increased metabolic activity of VAT promotes the release of free fatty acids into circulation, thereby inducing IR in adjacent tissues (216). A German population-based study employed quantitative MRI to evaluate the link between hepatic steatosis and prediabetes. Results showed that MRI-defined hepatic steatosis was significantly higher in individuals with prediabetes and positively correlated with several glucose metabolism markers, such as HbA1c, fasting glucose, and 2hPG (217). The underlying cause may be associated with IR induced by an accumulation of lipid metabolites in the liver (218), indicating that hepatic steatosis could serve as a potential biomarker for prediabetes. Bamberg et al. evaluated liver proton density fat fraction (PDFF) along with subcutaneous and visceral abdominal fat using MRI techniques. The findings indicated that PDFF levels, as well as total and visceral fat quantities, were significantly elevated in individuals with prediabetes, suggesting their potential role as indirect biomarkers (219). Borel et al. conducted simultaneous measurements of visceral fat and liver fat using CT imaging, revealing that liver fat content exhibited a positive correlation with isolated IGT (ilIGT), whereas visceral adiposity demonstrated a positive correlation with both ilIFG and ilIGT (220). Similarly, research measuring VAT volume through CT suggests it could serve as a reliable tool for assessing metabolic risk factors in individuals with prediabetes (221). Taking liver adipose tissue as an example, radiomics quantifies hepatic fat accumulation while metabolomics elucidates changes in metabolites associated with hepatic lipid metabolism, such as L-lactic acid and L-propionic acid (69). The integration of these two approaches not only facilitates the early identification of prediabetes risk but also enhances our understanding of its metabolic mechanisms, thereby providing a more precise foundation for personalized treatment and intervention.

Peripheral insulin sensitivity in humans largely depends on glucose utilization within skeletal muscle tissue. Consequently, alterations in skeletal muscle composition regarding fat content and lipid distribution may serve as critical determinants of IR (222). Kiefer et al. conducted a quantitative analysis of intracellular lipids (IMCLs) and extracellular lipids (EMCLs) in abdominal skeletal muscle using MRI, revealing that the levels of IMCLs and EMCLs in prediabetic patients were significantly elevated compared to those in normoglycemic control groups. This finding suggests that the distribution patterns of IMCLs and EMCLs may serve as promising biomarkers for prediabetes (223). Lipid metabolism abnormalities in skeletal muscle are closely associated with insulin resistance. Radiomics quantifies the distribution of IMCLs and EMCLs, revealing changes in lipid accumulation, while metabolomics analyzes shifts in metabolites such as glutamate, carnosine, and sphingomyelins, which are closely linked to lipid metabolism disturbances (74). The integration of these approaches provides a more comprehensive understanding of skeletal muscle metabolism, thereby facilitating the early identification and diagnosis of prediabetes.

Ectopic fat accumulation in the pancreas, known as pancreatic steatosis or fatty pancreas, increases the risk of prediabetes and diabetes (224). A fatty pancreas is typically characterized by hyperechoic pancreas (HP) when assessed via US. Research utilizing abdominal ultrasound has demonstrated that the risk ratio for glucose progression escalates with the severity of hyperechoic pancreatitis (225). Furthermore, moderate to severe HP serves as a reliable predictor for prediabetes and diabetes (226). Research has demonstrated that pancreatic fibrosis can lead to the destruction of pancreatic tissue, resulting in impaired secretion of pancreatic β cells and subsequent insulin deficiency (227). The hyperglycemic milieu induced by diabetes can lead to the aberrant activation of pancreatic stellate cells. These cells are capable of secreting a diverse array of pro-inflammatory and growth factors, which not only facilitate the synthesis of α-smooth muscle actin but also expedite the deposition of extracellular matrix components such as collagen, ultimately resulting in pancreatic fibrosis (228, 229). An extracellular volume fraction (fECV) derived from contrast-enhanced CT was employed to evaluate the extent of pancreatic fibrosis in prediabetic patients. The findings indicated that pancreatic fECV was significantly elevated in prediabetic individuals compared to non-diabetic counterparts and exhibited a moderate correlation with HbA1c levels (230). These findings suggest that pancreatic fECV could be a valuable biomarker for monitoring glucose dysregulation.

The investigation conducted by Askani et al. examined the correlation between adrenal volume measurements derived from MRI and impaired glucose metabolism. The findings revealed a significant increase in adrenal volume among prediabetic patients compared to healthy controls. However, this association weakened after adjusting for BMI (231). Therefore, adrenal volume could serve as an indirect biomarker of prediabetes, offering valuable insights for future research into its pathophysiology and the discovery of reliable biomarkers. Normally, perirenal fat appears hyperechoic or moderately echogenic on ultrasound imaging. In diabetes and prediabetes, IR and metabolic disorders often disrupt lipid metabolism and alter adipose tissue composition, resulting in a hypoechoic appearance on ultrasound. Shen et al. conducted an evaluation of 240 renal ultrasound cases and discovered that hypoechoic perinephric fat (HPF) demonstrated exceptionally high specificity and positive predictive value in patients diagnosed with prediabetes and diabetes (232). This finding indicates that HPF serves as a significant imaging biomarker closely associated with metabolic syndrome and IR.

Hyperechoic deltoid indicates that ultrasound imaging of the shoulder reveals an echo intensity greater than normal for this muscle. In individuals with diabetes or prediabetes, fatty infiltration and IR may lead to diminished glycogen levels within the deltoid muscle, resulting in a hyperechoic appearance. Research utilizing ultrasound assessments of the shoulder has demonstrated that the characteristic hyperechoic deltoid appearance serves as a robust predictor for prediabetes and can function as an adjunctive tool for early detection (233). The biomarkers associated with prediabetes, identified through radiomics analysis, are summarized in **Table 7**.

**Table 7:** Radiomics biomarkers associated with prediabetes.

Types of radiomics techniques	Source	Species	Biomarkers	Potential pathogenesis	Ref.
MRI	Adrenal gland	Human	Adrenal volume	The hypothalamic–pituitary–adrenal axis activation, IR	(231)
MRI	Liver	Human	Hepatic steatosis	IR	(217)
MRI	Abdominal skeletal muscle	Human	IMCLs, EMCLs	IR	(223)
MRI	Liver, abdominal fat	Human	Liver PDFF, abdominal fat quantities	IR, abnormal fat metabolism	(219)
CT	Pancreas	Human	Pancreatic fECV	Impaired secretion of pancreatic β cells	(230)
CT	VAT	Human	VAT volume	IR, abnormal fat metabolism	(221)
CT	Liver, VAT	Human	Liver fat and visceral adiposity	IR, abnormal fat metabolism	(220)
US	Perirenal fat	Human	HPF	IR	(232)
US	Pancreas	Human	HP	IR, impaired pancreatic β cell function	(225, 226)
US	Deltoid	Human	Hyperechoic deltoid	IR, Adipose tissue accumulation	(233)

Overall, insulin resistance is the most prevalent pathway implicated in radiomics-based biomarkers of prediabetes. Radiomics biomarkers, including adrenal volume, hepatic steatosis, IMCLs, EMCLs, liver PDFF, abdominal fat mass, VAT volume, HPF, HP, and hyperechoic deltoid, may be positively correlated with one another, working synergistically to promote the development of insulin resistance (217, 219, 221, 223, 225, 231–233). These biomarkers may interact through various mechanisms, such as influencing fat distribution, lipid accumulation, liver function, the endocrine role of adipose tissue, and local inflammatory responses, thereby contributing to the exacerbation and progression of insulin resistance.

#### 4.7.2 Limitations and future directions

Cross-sectional designs pose challenges in establishing causality, requiring future validation through larger cohort studies to confirm their clinical applicability (217). Many of these investigations have ambiguous underlying mechanisms, requiring further exploration for confirmation. Manual image segmentation is prone to subjective bias (231). However, integrating artificial intelligence (AI) into radiology could enable automated segmentation, facilitating data extraction from large datasets and identifying markers associated with disease risk profiles. Current radiomics research often lacks histopathological validation, and future investigations in this domain could be fortified to improve the robustness of their conclusions (230). Most imaging studies currently operate independently. Future efforts should integrate multiple imaging modalities to gain more comprehensive insights. Concurrently, the incorporation of advanced deep learning algorithms, such as convolutional neural networks and recurrent neural networks, is essential for enhancing diagnostic accuracy and predictive capabilities.

## 5 Multi-omics integration in prediabetes

Single omics studies concentrate on a singular type of biomolecule, often yielding a limited perspective (234). In contrast, multi-omics provides a more comprehensive understanding of disease pathogenesis and progression by analyzing gene expression, protein synthesis and modifications, and changes in metabolic products. Combining biomarkers from different omics approaches may generate a synergistic effect, enhancing diagnostic accuracy and offering deeper insights into disease mechanisms, compared to using individual markers alone. Furthermore, the integration of multi-omics data facilitates the identification of novel biomarkers, thereby providing new targets for disease diagnosis and treatment. Therefore, combining multi-omics with systems biology and bioinformatics tools is essential for achieving a holistic understanding of biological functions and the molecular mechanisms underlying diseases through cross-layer data integration (235, 236).

### 5.1 Biomarkers derived from multi-omics integration

Given the intricate upstream-downstream relationship between transcriptomics and proteomics, integrating them provides distinct advantages for disease research (236). Transcriptomic studies can reveal gene expression levels linked to disease states. However, transcription alone cannot confirm whether the corresponding proteins perform functional roles. Proteomics confirms the presence of these proteins and their disease-related alterations, providing more precise insights for identifying disease biomarkers. Moreover, transcriptomic and proteomic data complement each other (237). Sometimes, fluctuations in transcription levels are not reflected at the protein level due to post-transcriptional regulation, translational control, or protein degradation. Integrating these two datasets allows for collaborative learning, fostering a deeper understanding of regulatory mechanisms and enhancing biomarker reliability (238, 239). Belongie et al. conducted a comprehensive three-year cohort study focusing on plasma proteomics and miRNA to identify circulating biomarkers indicative of diminished β cell function and IGT. In the cross-sectional analysis conducted at year 3, adiponectin (ADPN), α1-AT, ESM-1, miR-181a, miR-342, and miR-323 exhibited the most pronounced differential expression as biomarkers in patients with IGT or a decline in β-cell glucose sensitivity. At baseline, the levels of ADPN, CTSD, and NCAM.L1—proteins produced by pancreatic β-cells—were significantly lower in individuals who progressed to IGT (240). These biomarkers effectively monitor β-cell function and predict future decline, positioning them as potential therapeutic targets for prediabetes.

Growing attention has been given to the interaction between intestinal microbiota and host metabolism, as both mutually influence each other and contribute to the pathophysiology of prediabetes. Following metagenomic sequencing and serum metabolomics analysis of gut microbiota, Zhang et al. discovered dysregulation in both the microbiome and metabolome among individuals with IGT, identifying numerous gut microbial taxa and metabolites that serve as predictors for the progression from IGT to diabetes (241).

Integrating imaging techniques and transcriptomic analysis provides a novel perspective in the search for prediabetes biomarkers. Chen et al. employed metabolic imaging techniques to visualize endogenous fluorescent compounds in adipose tissue, including reduced nicotinamide adenine dinucleotide (phosphate) [NAD(P)H], oxidized flavin adenine dinucleotide (FAD), and lipofuscin-like pigments, thereby achieving non-invasive visualization of the metabolic characteristics of macrophages and adipocytes within adipose tissue. This methodology enables the localization of macrophages and has revealed that only adipocytes within the adipose tissue of prediabetic individuals exhibit specific metabolic fluorescence alterations, such as a low oxidation-reduction ratio and an elevated free NAD(P)H fraction. Furthermore, RNA-Seq analysis revealed alterations in the expression of several genes associated with oxidative metabolism in adipose tissue of prediabetic individuals, including the downregulation of PGC-1α and ETC genes (242). This finding aligns with the metabolic imaging observations of changes in adipocyte metabolism, further substantiating the correlation between metabolic fluorescence variations and gene expression. Consequently, alterations in adipocyte metabolic fluorescence are anticipated to serve as potential biomarkers or risk factors for IR in prediabetic individuals. **Table 8** summarizes the prediabetes-associated biomarkers identified via multi-omics integration.

**Table 8:** Biomarkers derived from multi-omics integration.

Omics types	Source	Species	Biomarkers	Potential pathogenesis	Ref.
Proteomics, transcriptomics	Plasma	Human	ADPN↓, α1-AT↓, ESM-1↓, CTSD↓, NCAM.L1↓ miR-181a↓, miR-342-3p↓, miR-323-3p↓	Epithelial-mesenchymal transition, β cells dysfunction	(240)
Microbiomics, metabolomics	Gut microbiota, serum	Human	Eggerthella unclassified↑, Coprobacillus unclassified↑, Clostridium ramosum↑, Eubacterium eligens↓, Bacteroides faecis↓, Lachnospiraceae bacterium 3_1_46FAA↓, Alistipes senegalensis↓, Megaspaera elsdenii↓, Clostridium perfringens↓, L-valine↑, L-norleucine↑, L-isoleucine↑, α-linolenic acid↓, 10E↓,12Z-octadecadienoic acid↓, dodecanoic acid↓	–	(241)
Radiomics, transcriptomics	Adipose tissues	C57BL/6 mice	Alterations in adipocyte metabolic fluorescence, PGC-1α↓, ETC↓	IR, mitochondrial dysfunction, alteration in metabolic pathways	(242)

ESM-1, endocan; α1-AT, alpha-1-antitrypsin; CTSD, cathepsin D; NCAM.L1, neural cell adhesion molecule ligand 1; PGC-1α, peroxisome proliferator-activated receptor gamma coactivator 1-alpha; ETC, electron transport chain; “–” The information has not been obtained from the primary publication or has not been found yet.“↑” The symbol indicates upregulation of the marker in prediabetes. “↓” The symbol indicates downregulation of the marker in prediabetes.

### 5.2 The challenges and future directions of multi-omics technology

Integrating multi-omics to identify novel biomarkers is a rapidly evolving and promising field. However, several challenges remain, including data quality, standardization, noise interference, reproducibility issues, and validation processes (234). High-throughput technologies generate exceptionally large datasets, placing significant demands on computational resources. A promising trend is the integration of multi-omics data analysis with AI and machine learning. This approach is expected to enhance the predictive power and accuracy of multi-omics research, facilitating the development of precise disease models and personalized therapies (243, 244). Additionally, access to diverse omics databases and analytical tools provides researchers with extensive options to analyze and interpret complex omics data, enabling deeper insights into the biological mechanisms underlying disease (245).

## 6 Conclusion and perspective

Timely identification of prediabetes and prompt intervention are essential to halt the progression of diabetes. Although some biomarkers overlap between prediabetes and diabetes, the two conditions are not fully congruent. This observation suggests that prediabetes may represent a distinct disease stage, thereby facilitating independent investigation of its diagnostic markers (111). Molecular, cellular, and tissue changes occur during prediabetes. Recent progress in high-throughput methodologies has provided a distinct opportunity to explore the complex connections among different histological and phenotypic targets (246). Utilizing multi-omics technology allows systematic investigation of effector molecule variation across levels, deepening our understanding of biomolecular interactions and their impact on functional and phenotypic traits. This approach offers robust theoretical and data support for uncovering mechanisms regulating prediabetes. The biomarkers identified through multi-omics technology can partially compensate for the limitations of traditional screening indicators and significantly aid in the screening, diagnosis, and management of prediabetes.

Although numerous predictive biomarkers for prediabetes have been identified, the sensitivity and specificity of most remain poorly understood, and their underlying mechanisms are not fully elucidated (53, 118, 124). The majority of biomarkers are identified from clinical samples and hold practical significance in clinical applications. However, some biomarkers derived from animal samples may exhibit limited reliability when applied to humans (57, 67, 153, 172). To translate potential biomarkers into clinically applicable diagnostic tools, it is essential to first validate their sensitivity, specificity, and applicability through large-scale clinical studies. Concurrently, efforts should be made to streamline the detection process and develop low-cost, high-throughput platforms to facilitate widespread implementation. Rigorous quality control and robust statistical methodologies are crucial to ensure the reliability and reproducibility of the biomarker findings (247, 248). Ultimately, these biomarkers must undergo regulatory approval and satisfy clinical standards before they can be effectively integrated into disease screening, diagnosis, and management (249).

Current findings suggest that combining biomarkers to form predictive models could enhance the diagnostic predictive capability for prediabetes (73). Future research could integrate omics biomarkers with traditional clinical indicators (such as BMI, blood pressure, and lipid profiles) to develop multi-dimensional predictive models, thereby enhancing the accuracy and predictive power of early prediabetes diagnosis. Such comprehensive models would not only improve early detection of disease onset but also offer better adaptability to the clinical needs of diverse populations, thereby increasing their potential for widespread application. Omics data should be integrated into a unified framework to advance precise diagnosis (250, 251). Specifically, this can be achieved by establishing cross-omics data integration platforms, standardizing the data, applying machine learning algorithms for data fusion, and incorporating clinical validation to assess the clinical relevance of biomarkers (252). Moreover, the development of visualization tools and decision support systems will enhance diagnostic accuracy and improve clinical applicability. It is worth noting that many prediabetic patients may already have complications (253, 254), but current biomarkers rarely predict these conditions. When evaluating the predictive capacity of biomarkers for prediabetes, it is crucial to consider their ability to forecast long-term risks. Future studies should investigate the role of these biomarkers in assessing the long-term risk of progression from prediabetes to diabetes, as well as their potential in monitoring the effects of lifestyle interventions or pharmacological treatments on the disease trajectory.

In conclusion, multi-omics technologies hold considerable promise in providing novel insights into prediabetes. Biomarkers can play a pivotal role in personalized treatment by enabling the comprehensive assessment of a patient’s metabolic, immune, and genetic profiles, which can inform the development of precision therapeutic strategies (255, 256). Based on the specific biomarker levels of individual patients, clinicians can tailor interventions, such as pharmacological treatments or lifestyle modifications, to optimize outcomes. With advancements in machine learning and artificial intelligence, the integration of multi-omics data could facilitate the creation of personalized treatment models, allowing for more customized therapeutic approaches and enhancing the application of precision medicine in prediabetes management (257).
